# Impact of tDCS on working memory training is enhanced by strategy instructions in individuals with low working memory capacity

**DOI:** 10.1038/s41598-021-84298-3

**Published:** 2021-03-09

**Authors:** Sara Assecondi, Rong Hu, Gail Eskes, Xiaoping Pan, Jin Zhou, Kim Shapiro

**Affiliations:** 1 Visual Experience Laboratory, School of Psychology, University of Birmingham, Birmingham, UK; 2 Center for Human Brain Health (CHBH), University of Birmingham, Birmingham, UK; 3 Department of Neurology, Guangzhou First People’s Hospital, School of Medicine, South China University of Technology, Guangzhou, Guangdong China; 4 Departments of Psychiatry and Psychology & Neuroscience, Dalhousie University, Halifax, NS Canada

**Keywords:** Working memory, Cognitive neuroscience

## Abstract

Interventions to improve working memory, e.g. by combining task rehearsal and non-invasive brain stimulation, are gaining popularity. Many factors, however, affect the outcome of these interventions. We hypothesize that working memory capacity at baseline predicts how an individual performs on a working memory task, by setting limits on the benefit derived from tDCS when combined with strategy instructions; specifically, we hypothesize that individuals with low capacity will benefit the most. Eighty-four participants underwent two sessions of an adaptive working memory task (n-back) on two consecutive days. Participants were split into four independent groups (SHAM vs ACTIVE stimulation and STRATEGY vs no STRATEGY instructions). For the purpose of analysis, individuals were divided based on their baseline working memory capacity. Results support our prediction that the combination of tDCS and strategy instructions is particularly beneficial in low capacity individuals. Our findings contribute to a better understanding of factors affecting the outcome of tDCS when used in conjunction with cognitive training to improve working memory. Moreover, our results have implications for training regimens, e.g., by designing interventions predicated on baseline cognitive abilities, or focusing on strategy development for specific attentional skills.

## Introduction

Non-invasive direct current brain stimulation (tDCS) can improve performance on a variety of cognitive tasks by exploiting mechanisms of synaptic long-term potentiation and depression ^1,2^. Crucially, these mechanisms are particularly efficient when the stimulated area is involved in the cognitive processes under investigation ^1,3^. An interesting application of this principle is in the context of working memory. Working memory (WM) is a core cognitive function that has been linked to many facets of human cognition, such as attention, memory, language, and general intelligence ^4^. WM plays an important role in many aspects of everyday life ^5^ but, critically, is a limited capacity system ^6–8^ that declines with age ^9^ and is compromised by several pathologies, such as epilepsy, schizophrenia, Alzheimer’s disease, mild cognitive impairment, and brain injury ^10^.

Researchers have examined interventions to improve working memory, e.g. by combining task rehearsal and non-invasive brain stimulation ^11^. Approaches have included both single- and multi-session designs, with brain stimulation applied concurrently or before a cognitive task and have examined both healthy as well as clinical populations. In spite of its potential, however, outcomes of these studies are inconsistent and highly dependent on methodological parameters (for a review, see ^12^). When multisession designs are considered, results are even less consistent across studies. For example, whereas an improvement in working memory performance was reported when combining multiple sessions of working memory training with tDCS in healthy young volunteers ^13–15^, Nilsson et al. ^16^ found no evidence of improvement in the young or in the elderly. Thus, researchers call for caution in interpreting the impact of tDCS on working memory, hence one of the motivations of the present study ^17–19^.

Many factors have been shown to affect the outcome of studies examining the use of tDCS to improve WM, as measured by the n-back task. In the n-back task, participants are requested to decide if a stimulus in a sequence matches the one appeared ‘n’ items before ^20^. Gill et al. ^21^ showed that a 3-back working memory task but not a 1-back task led to a poststimulation performance improvement, and others have stressed that the targeted brain network, usually the DLPFC in working memory studies, should be engaged in a cognitive task to maximize the influence of brain stimulation ^12,22–24^. Others have suggested ceiling and floor effects should be avoided ^25,26^, that the location of the electrodes on the scalp should be congruent with the brain areas engaged in the working memory task ^27–29^, and that individual differences in age and education influence performance ^30,31^. As Berryhill^32^ pointed out, most of the inconsistency in combined working memory training and tDCS experiments can be attributed to heterogeneity in parameters and experimental designs.

Importantly and relevant to the present study, the choice of a specific strategy applied to a cognitive task has been shown to exert a significant impact ^33–35^. In working memory training regimens, participants who develop a strategy early in a cognitive task are more likely to achieve better overall training task performance than those who fail to derive a consistent or useful strategy. The rationale offered is that providing strategy instructions reduces differences in how initial skill sets are used, compensating for the cognitive limitations of those less equipped to fully exploit their resources, and potentially unveiling individual differences in cognitive plasticity ^35^. However, the impact of strategy on multi-session design has only recently been addressed ^36^, showing that a strategy advantage is present only in the first few sessions of training. The impact of combining tDCS and strategy instructions in WM training regimens is yet to be fully investigated and serves as the motivation for the present study. Thus, our goal was to examine the benefit of combining tDCS and strategy instructions in young adults performing two sessions of a working memory training task. Additionally, we hypothesized that working memory capacity at baseline predicts how an individual performs on a working memory task by setting limits on the benefit derived from tDCS combined with strategy instructions.

## Material and methods

### Participants

Ninety-two (65 female) right-handed participants (mean age = 20.6 ± 3.8, range 18 to 39) were recruited from the University of Birmingham or community and were compensated for their time (3 h in total) with either course credits or £20. Participants who did not fulfil safety inclusion criteria for brain stimulation ^37^, had a history of depression, or had received brain stimulation or cognitive training in the previous 6 months were not eligible for the study. Eight participants dropped out after the first day, resulting in a total of 84 participants. The research procedures were approved by the University of Birmingham’s Ethics Committee and are in accordance with the Declaration of Helsinki. All participants gave their informed consent before starting the study.

Participants were randomly assigned to four groups, ACTIVE and SHAM receiving either active or sham stimulation and crossed with STRATEGY and NOSTRATEGY, either receiving strategy instructions, or not receiving strategy instructions. We obtained four groups: ACTIVE-STRATEGY, ACTIVE-NOSTRATEGY, SHAM-STRATEGY, SHAM-NOSTRATEGY. The SHAM-NOSTRATEGY acts as control group, as participants in this group received no intervention (either stimulation or strategy). We employed an adaptive spatial n-back paradigm (aNback), over two sessions, with concurrent tDCS of the right DLPFC (online). We evaluated the impact of strategy and stimulation by comparing task performance from pre- to a post- session (offline, e.g., without tDCS) on the same adaptive spatial n-back and on a fixed-load visual n-back (fNabck), to tease apart the effects of brain stimulation and strategy development.

### Working memory capacity scores

As we predicted a relationship between baseline performance and outcome of the intervention, at the analysis stage we divided participants according to their composite memory capacity score at baseline. For each participant, a composite capacity score was calculated as the mean of the standardized scores of the two tasks completed at baseline (the adaptive spatial Nback (aNback) and the fixed load visual Nback (fNabck)). The scores for the aNback (e.g., the average value $\overline{n}$ across the baseline session) and for the fNback (e.g. the average of d-prime for n = 2 and n = 3 at baseline) were standardized as follows: DVz = (DV-$\mu$)/$\sigma$, where DVz is the standardized dependent variable, DV is the original dependent variable, $\mu$ and $\sigma$ are the mean and standard deviation of the DV over the entire sample (84 participants). At the analysis stage, participants were then divided into high- (above 66th percentile), mid- (between 33rd and 66th percentile) and low-capacity (below 33rd percentile) levels. When using baseline performance to split participant data before analysis, there is a risk of incurring regression to the mean, in turn confounding treatment effects ^38–40^. To avoid this issue, we have taken the following steps: first we used a combination of two baseline measures to calculate the composite scores, and secondly in each capacity level we included a control group (receiving neither stimulation nor strategy training). This approach allows us to safely carry out group comparisons within CAPACITY levels, as well as comparison of differential effects (TREATMENT–CONTROL) between CAPACITY levels. At the same time, we avoid comparing absolute effects between LOW and HIGH CAPACITY individuals, which are prone to regression-to-the-mean effects.

### Transcranial direct current stimulation

Transcranial direct current stimulation (tDCS) was administered using an eight-channel device (Starstim, Neuroelectrics). Participants received stimulation during two practice sessions over two consecutive days via two circular Ag/AgCl electrodes (NG Pistim, Neuroelectrics) of 1 cm radius (3.14cm^2^ area). Electrode impedance was kept below 10 kOhm by using a conductive gel (SignaGel, ParkerLabs) between electrodes and scalp. The anode^41^ was placed over the right dorsolateral prefrontal cortex (rDLPFC, F4^29,42^) with the cathode over the contralateral supraorbital site (Fp1), according to the international 10–20 system. In the ACTIVE group the current was ramped up to 2 mA (current density = 0.64 mA/cm^2^) in the first 30 s and maintained for 20 min before ramping down to 0 mA in the last 30 s (total ACTIVE time 21 min). In the SHAM group current was ramped up to 2 mA in the first 30 s then immediately ramped down to 0 mA in the next 30 s, where it was maintained for 20 min followed by another cycle of ramping up and down (total SHAM time 21 min). Participants were randomly assigned to either the ACTIVE or the SHAM tDCS condition and both blinding and side effects were monitored via a feedback questionnaire.

### Adaptive spatial nback task (aNback)

**Figure 1:**
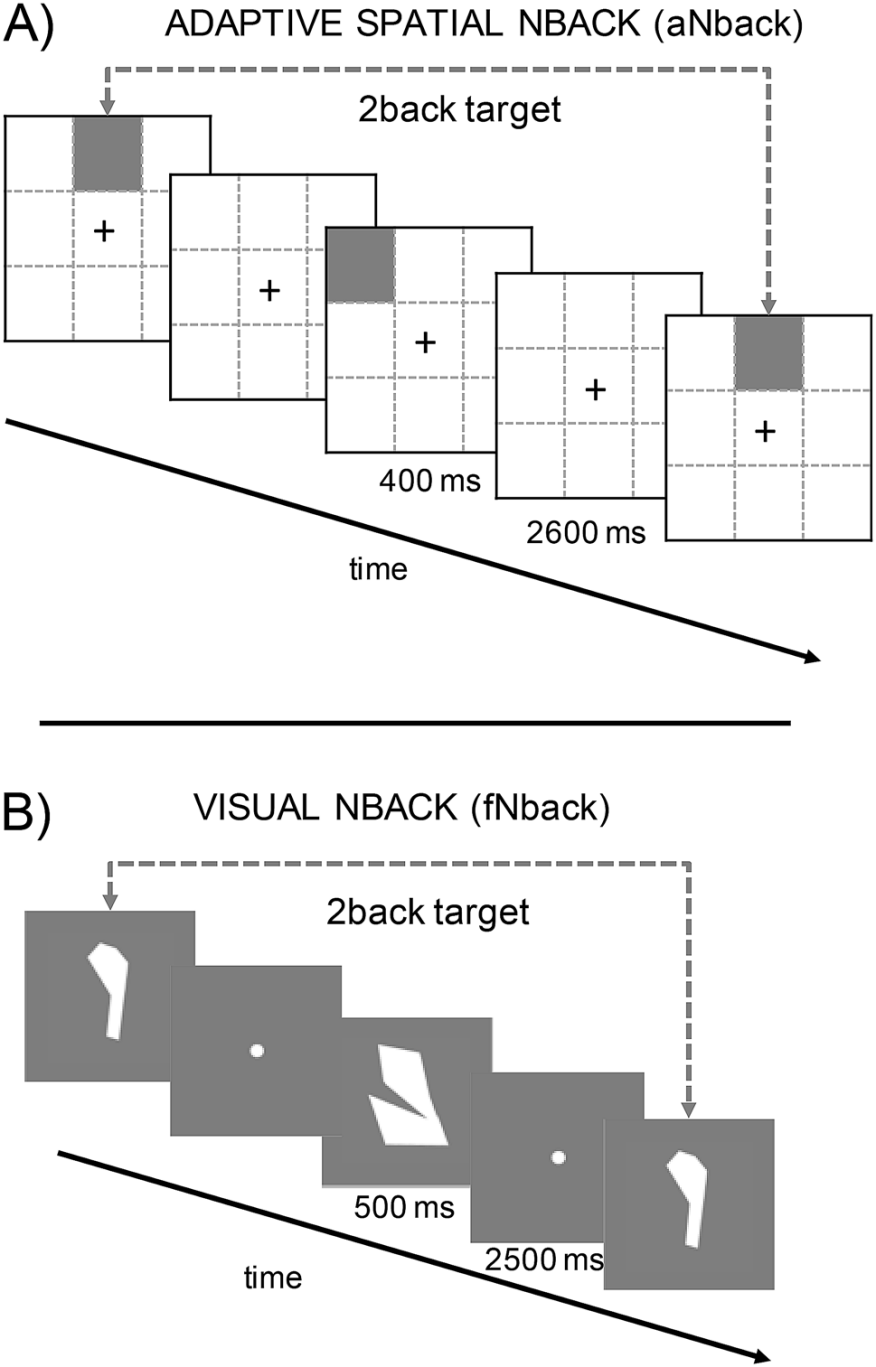
Description of the two tasks used in the experiment. (**A**) illustrates the adaptive Nback training task (aNback), whereas (**B**) depicts the fixed Nback outcome task (fNback).

In the adaptive N-back working memory task (aNback, see Fig. 1, Panel A) participants were told to monitor the position of a sequence of blue squares appearing randomly in one of the eight positions defined by a 3 × 3 grid on the screen (centre position excluded), and to report whether the current location matched a previous one ‘n’ trials ago (Brain Workshop 4.8.7, http://brainworkshop.sourceforge.net/). Each square was presented for 0.4 s, followed by an empty grid for 2.6 s (total trial length 3 s). Participants were asked to be as accurate and as fast as possible, responding to match (target) trials by pressing the key “A”, whereas non-match (non-target) trials did not require a response. Participants received feedback on their accuracy every time a response was given. Starting from a difficulty of n = 2 (indicating ‘n’ items to remember), as participants' performance score (True Positives/(True Positives + False Positive + False Negatives)) was at or above 70% in a block, difficulty would increase by one, or decrease by one if it was below 50%. Each training session consisted of 15 blocks or sequences of 20 + n trials each. Each block of trials included 12.5% matches and 12.5% interference (e.g., 12.5 of non-match trials presented the target item at position n-1 or n + 1 instead of n). At the end of each block participants received feedback on performance and the task was appropriately adapted for the subsequent block.

### Fixed-load visual nback task (fNback)

Participants’ visual working memory was assessed on a nonadaptive visual NBACK task with random shapes (fNback, see Fig. 1, Panel B)^43^. Stimuli consisted of ten random shapes presented sequentially in the centre of the screen for a total of 0.5 s, followed by a fixation cross for 2.5 s (total trial length 3 s). Participants were asked to be as accurate and as fast as possible while searching for shapes matching with ‘n’ items before, responding by pressing “A” for matches and “L” for no matches. Participants completed six blocks, or sequences, for n = 2 and six blocks for n = 3, for a total of 120 trials for each n. Each sequence included 30% matches and 10% interference (as defined above).

### Strategy instructions and questionnaires

**Figure 2:**
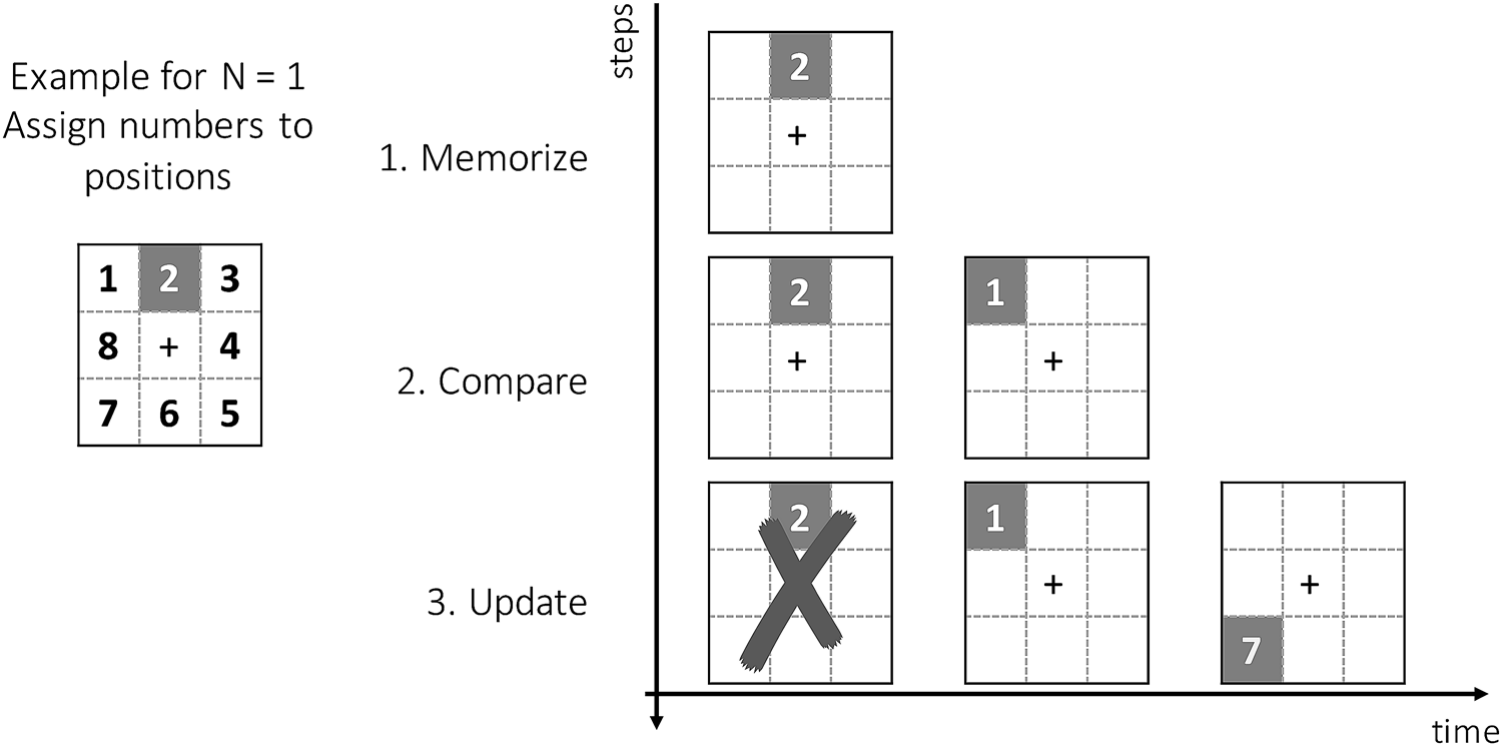
Schematic of strategy instructions. Participants were asked to assign numbers to positions on the spatial grid, then to create in their mind a target set (grouping) of the positions as numbers as the first ‘n’ items are presented, then compare the new item on the screen with the appropriate recent item (depending on n level) in their memory set, and finally to discard the least recent item of the sequence in mind and update the target set with the new item. Participants in the no- strategy group were introduced to the task and instructed to “do their best trying to get to the highest possible n level”.

Before starting the first session with concurrent tDCS (ACTIVE or SHAM) and the adaptive nback training task, half of the participants were provided with clear instructions on how to undertake the WM task. The strategy follows Laine et al. ^33^, and is depicted in Fig. 2. Participants were first asked to associate a number to each of the eight positions on the screen and were reminded that the central position was unavailable. Then they were briefed on the use of a strategy based on “memorize’, ‘compare’ and ‘update’ (Fig. 2). After the last session, participants completed a questionnaire reporting on the strategy used (see Supplemental Material).

Mood and motivation were monitored by administering the Positive Affect/Negative Affect Schedule (PANAS ^44^) at the beginning of the baseline session and at the end of the post-assessment, in addition to 5 additional questions on a Likert scale (1 to 5, on alertness, motivation, sadness and expectation on both the WM performance and the effect of tDCS) to be answered based on one’s subjective ‘feeling’ before each administration of the adaptive Nback task.

### Procedure

**Figure 3:**
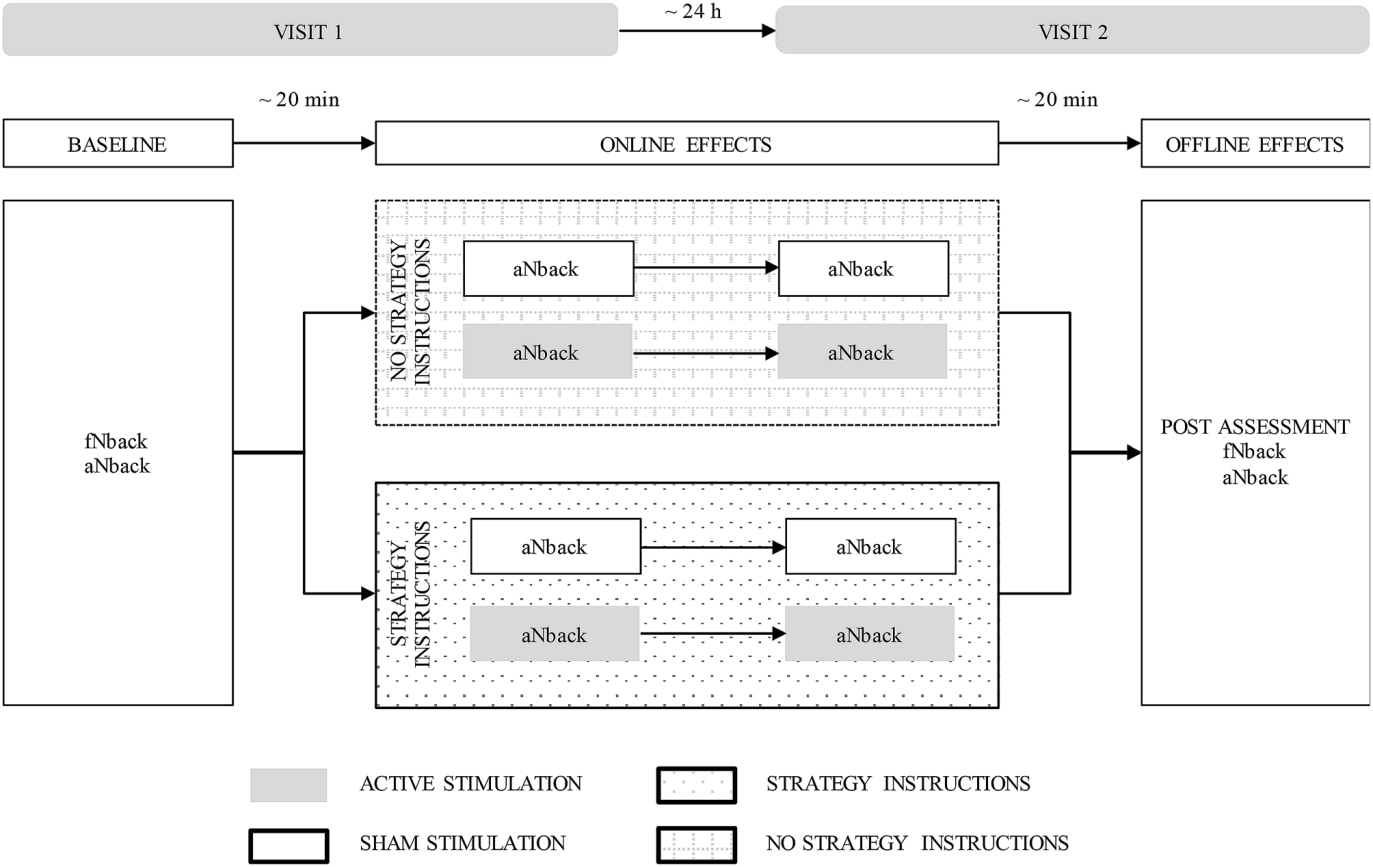
Experiment’s timeline.

The timeline of the experiment is shown in Fig. 3. Participants completed the study over two visits on two consecutive days. During the first visit, all participants underwent the baseline assessment, in the following order: PANAS, one session of the fNback, and one session of the aNback. The STRATEGY group received detailed instructions on a specific strategy proven to be task beneficial, while those in the NoSTRATEGY group were not instructed to develop a strategy. Both groups were encouraged to achieve as high an ‘n’ as possible. Following the administration of strategy instructions (according to the respective group), participants were prepped for brain stimulation and undertook another session of the aNback with either SHAM or ACTIVE tDCS, which completed the first visit. On the second visit the following day, participants first completed a session of the aNback with tDCS (ACTIVE or SHAM), followed by the post-assessment including in the following order: one session of the aNback (without tDCS), one session of the fNback, and the PANAS. The two tDCS sessions provided data on the online effects of combining tDCS with strategy instructions, whereas offline effects were quantified during the post-assessment. Before each session of the aNback task, participants completed a motivation and expectation questionnaire, and a feedback form on the side effects of tDCS after each brain stimulation session (both ACTIVE and SHAM). At the end of the second visit they also completed a feedback form on the strategy used during both tasks (see Supplemental Material) and answered some questions about the blinding.

### Analysis

Our analysis of variance used a between-subject design with STIMULATION (2 levels), STRATEGY (2 levels) and CAPACITY (3 levels) as independent factors. Details of each analysis are provided in the results section, before describing the corresponding results. Greenhouse–Geisser sphericity correction was used, when necessary, as well as Holm correction for multiple comparisons in post-hoc tests. Finally, for the different CAPACITY levels, we report the model that best explain the data, together with its associated Bayes Factor in support of the alternative hypothesis (BF_10_).

## Results

### Initial baseline data

A 1-way independent ANOVA showed that the four groups did not differ in age, gender distribution, years of education, motivation, mood, attitude or performance in the baseline session of the fNback and the aNback (all ps > 0.05, see Supplementary Material). A chi-square test of independence showed no significant association between actual and perceived stimulation, indicating that subjects were blind to the stimulation group (X^2^_(1, N=84)_ = 0.86, p = 0.35), see Supplemental Material for tabulated values.

### Working memory capacity scores

**Figure 4:**
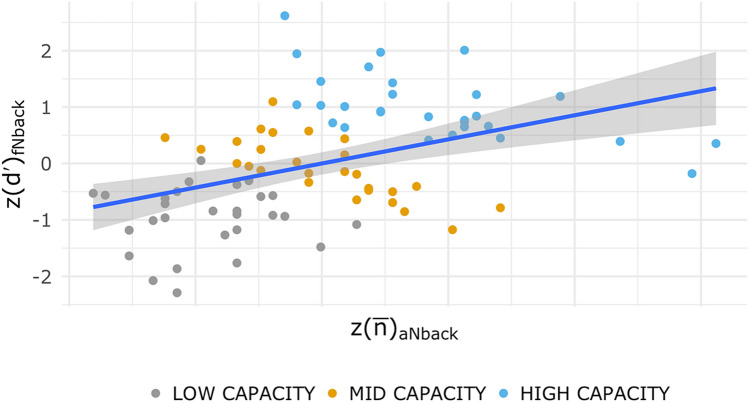
Correlation plot between standardised aNback (x-axis) and fNback (y-axis) scores at baseline. Points are color-coded according to their capacity group membership.

Scores for the two tasks were significantly related but shared only a small amount of variance (r_p_ = 0.43, p < 0.001, see Fig. 4). Descriptive statistics of the capacity scores are reported in the Supplementary Table S1. No differences in capacity scores were found between the four groups, when the capacity levels are aggregated (STIMULATION × STRATEGY, p > 0.1). There was no significant association between the groups and the capacity membership (X^2^(6) = 2.79, p = 0.84). Thus, there were no factors compromising the results of the independent variables under investigation.

### Online effects of the intervention

To evaluate the online effect of tDCS and strategy instructions, we quantified performance changes in training across the two tDCS sessions in the adaptive spatial nBack task (aNback) as the average difference between the mean ‘n’ within a session (excluding the first block) and the mean ‘n’ at baseline ($\Delta \overline{n} = \overline{n} - \overline{n}_{baseline}$). We predicted that the combination of tDCS and strategy instructions would be particularly beneficial in the LOW CAPACITY group. To test this prediction, we conducted a 4-way mixed ANOVA with 3 between-subject factors (STIMULATION: ACTIVE, CONTROL × STRATEGY: STRATEGY, NoSTRATEGY × CAPACITY: LOW, MID, HIGH) and one within-subject factor (TIME: change at DAY 1, DAY 2). We found a main effect of TIME (F_(1,72)_ = 35.08, p < 0.001, $\eta_{p}^{2}$ = 0.33) and STRATEGY (F_(1,72)_ = 4.80, p = 0.03, $\eta_{p}^{2}$ = 0.06), and significant interactions of TIME × STRATEGY (F_(1,72)_ = 5.47, p = 0.02, $\eta_{p}^{2}$ = 0.07) and STIMULATION × STRATEGY × CAPACITY (F_(2,72)_ = 5.83, p = 0.005, $\eta_{p}^{2}$ = 0.14). We then focused on the higher level 3-way interaction, after collapsing across TIME and conducted multiple comparisons between groups (t-test, Holm-corrected), within each CAPACITY level (LOW, MID, HIGH). The best model that describes the data included a main effect of STRATEGY, STIMULATION and their interaction (for LOW CAPACITY individuals, BF_10_ = 6.34, for HIGH CAPACITY individuals BF_10_ = 1.97).

**Figure 5:**
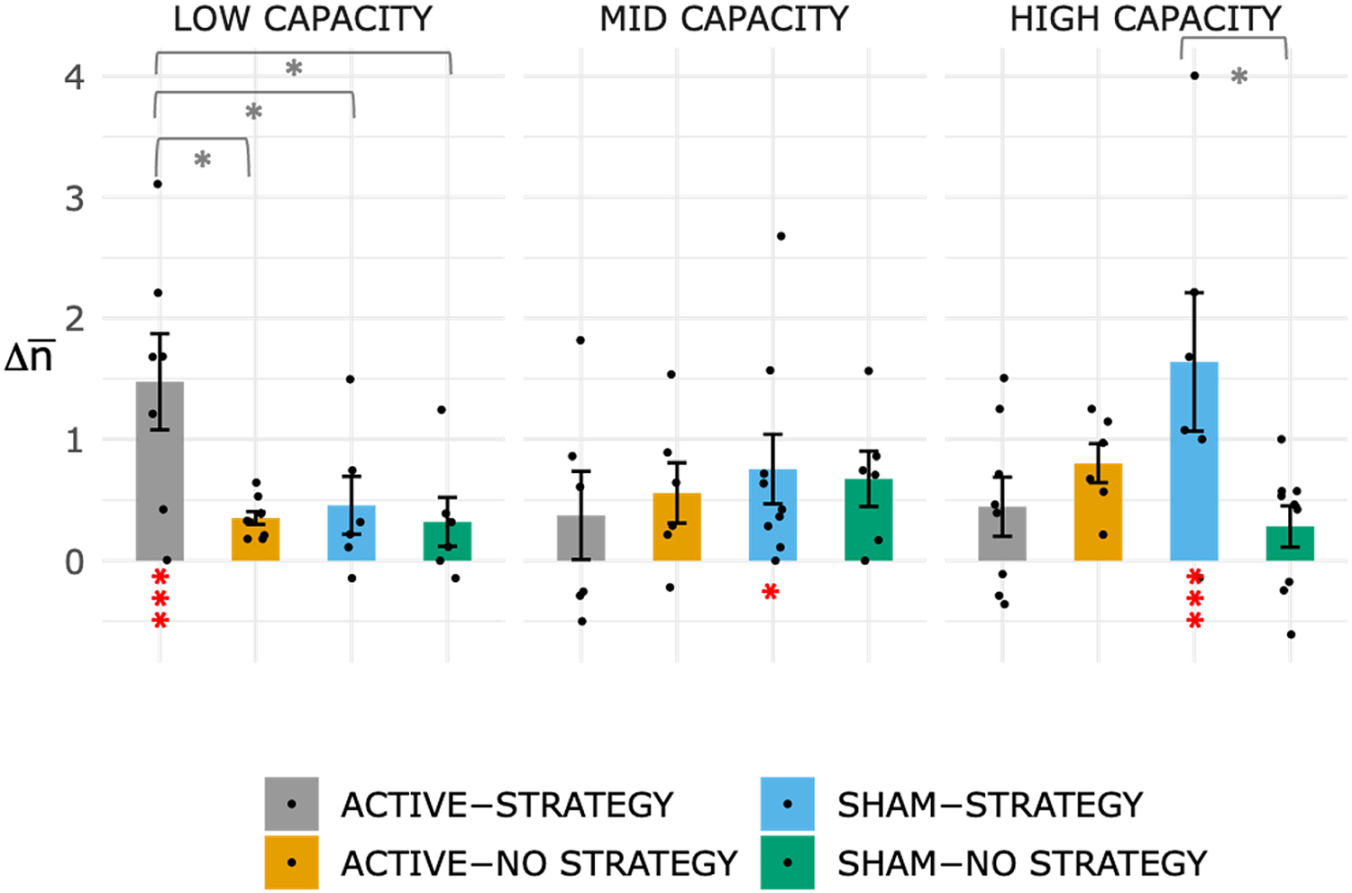
Performance change ($\Delta \overline{n}$) with respect to baseline, collapsed across day 1 and day 2 (tDCS sessions) for each group. Means with standard errors are reported for each group and capacity. P-values are marked as follows: * p < 0.05, ** p < 0.01, *** p < 0.001. Changes significantly larger than zero are marked in red.

Within low-capacity individuals, only the ACTIVE-STRATEGY group achieved performance improvements significantly larger than zero (t_(24)_ = 6.10, p < 0.001), and larger than ACTIVE-NoSTRATEGY (t_(14)_ = 3.48, p_H_ = 0.01, d_Cohen_ = 1.60) , SHAM-STRATEGY (t_(11)_ = 2.86, p_H_ = 0.03, d_Cohen_ = 1.16), and SHAM-NoSTRATEGY (t_(11)_ = 3.24, p_H_ = 0.02, d_Cohen_ = 1.36). No significant differences between groups were found in individuals with mid-capacity (ps > 0.1). For high-capacity individuals, SHAM-STRATEGY group’s improvement was significantly greater than zero (t_(25)_ = 5.02, p < 0.001) and larger than the SHAM-NoSTRATEGY group (t_(13)_ = 3.22, p_H_ = 0.021, d_Cohen_ = 1.42). Figure 5 shows performance changes, collapsed across the two online sessions (TIME), with respect to baseline, split by CAPACITY.

### Offline effects of the intervention

**Figure 6:**
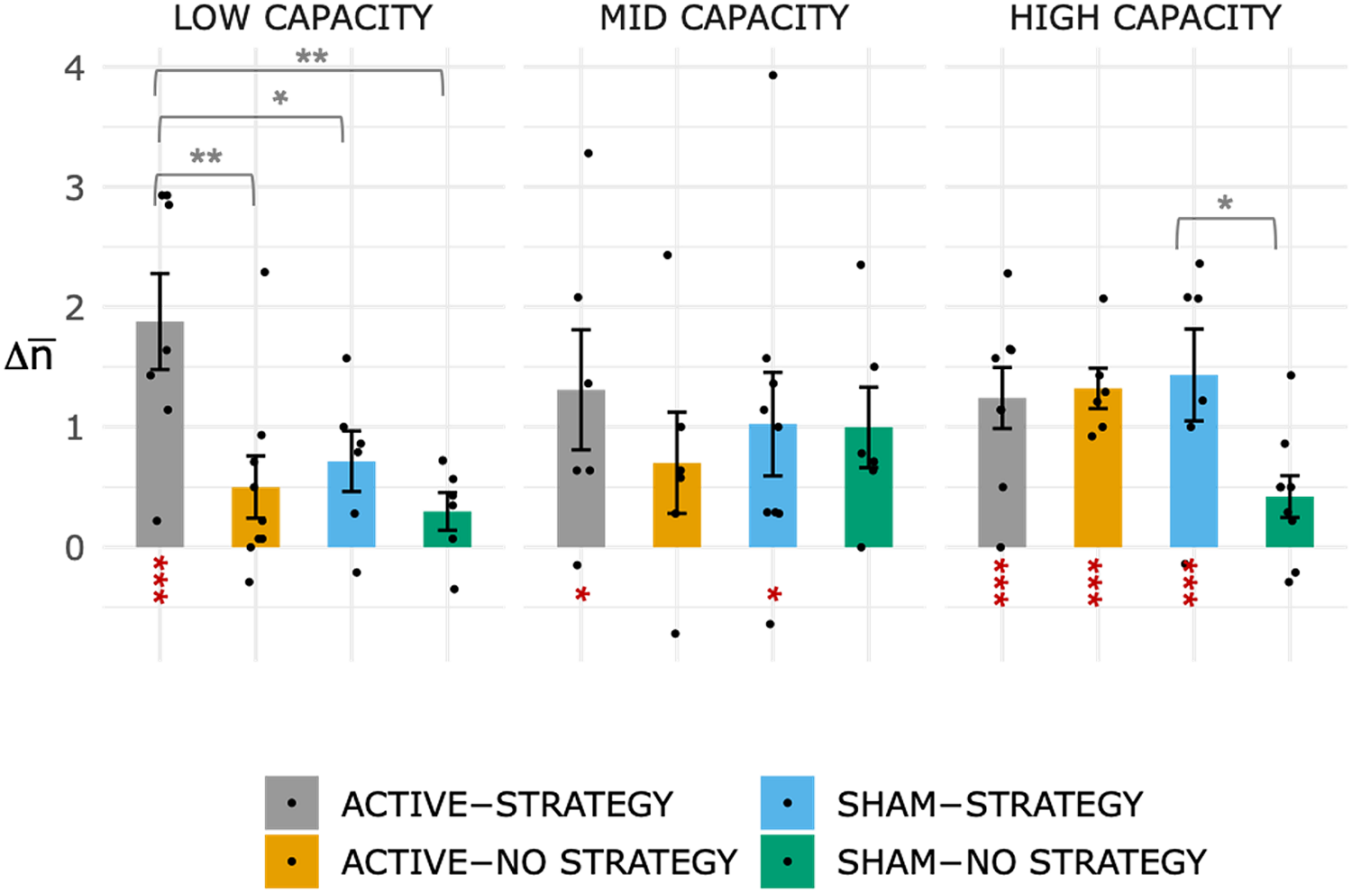
Increase in mean ‘n’ ($\Delta \overline{n}$) on the aNBack at the offline POST-ASSESSMENT with respect to BASELINE: means with standard errors are reported for each group and capacity. P-values are marked as follows: * p < 0.05, ** p < 0.01, *** p < 0.001 Improvement significantly larger than zero are marked in red.

To examine the overall effects of the intervention on working memory (offline effects) after two sessions of brain stimulation, we analysed the changes in performance in the aNback task ($\Delta \overline{n}$), calculated as the change in $\overline{n}$ in the post-assessment in relation to the baseline. Again, we predicted the combination of tDCS and strategy instructions would be particularly beneficial in the low-capacity group. A 3-way independent ANOVA (STIMULATION: ACTIVE, CONTROL × STRATEGY: STRATEGY, NoSTRATEGY × CAPACITY: LOW, MID, HIGH) revealed a significant main effect of STRATEGY (F_(1,72)_ = 8.40, p = 0.005, $\eta_{p}^{2}$ = 0.10) with the STRATEGY group achieving larger improvement than the NO STRATEGY group (Dc = 0.63), and a main effect of STIMULATION trending towards significance (F_(1,72)_ = 3.19, p = 0.08, $\eta_{p}^{2}$ = 0.04), with the ACTIVE group achieving larger performance improvements than the SHAM group (Dc = 0.37). We also found a 3-way interaction of STIMULATION × STRATEGY × CAPACITY trending towards significance (F_(2,72)_ = 2.71, p = 0.07, $\eta_{p}^{2}$ = 0.07). The best model that describes the data includes a main effect of STRATEGY, STIMULATION and their interaction (for LOW CAPACITY individuals, BF10 = 11.07, for HIGH CAPACITY individuals BF10 = 2.32). As our hypothesis is specific to individuals with low-capacity and the effect size of the 3-way interaction is medium to large ^57,58^, we followed up the 3-way interaction with planned multiple comparisons between groups (t-test, Holm-corrected), within each CAPACITY level (LOW, MID, HIGH). In individuals with low-capacity, the ACTIVE-STRATEGY group achieved offline performance improvements significantly larger than zero (t_(24)_ = 6.47, p < 0.001) and larger than the other 3 low-capacity groups (ACTIVE-NoSTRATEGY: t_(14)_ = 3.56, p_H_ = 0.008, d_Cohen_ = 1.52; SHAM-STRATEGY: t_(11)_ = 2.72, p_H_ = 0.05, d_Cohen_ = 1.31; SHAM-NO STRATEGY: t_(11)_ = 3.70, p_H_ = 0.007, d_Cohen_ = 1.91). No significant differences between groups were found within individuals with mid-capacity (ps > 0.1). In individuals with high-capacity, we found offline performance changes significantly larger than zero in the ACTIVE-STRATEGY (p < 0.001, t_(25)_ = 5.28), the ACTIVE-NoSTRATEGY (p < 0.001, t_(25)_ = 4.87) and the SHAM-STRATEGY (p < 0.001, t_(25)_ = 5.26), while only the SHAM-STRATEGY group achieved improvements in performance significantly larger than the SHAM-NoSTRATEGY group (t_(13)_ = 2.88, p_H_ = 0.049, d_Cohen_ = 1.42). Figure 6 shows performance changes at session 4 with respect to baseline, split by CAPACITY.

To establish if any benefit acquired with the optimal combination of tDCS and strategy instructions transferred to a different working memory task, we evaluated post-assessment performance changes with respect to baseline in the fNback task. After excluding the first block from the analysis, for each ‘n’ (n = 2 and n = 3) we calculated d-prime as a measure of performance (d-prime = z(Hits)-z(False Alarm) ^45,46^). A 3-way independent ANOVA with 3 between-subject factors STIMULATION: ACTIVE, CONTROL × STRATEGY: STRATEGY, NoSTRATEGY × CAPACITY: LOW, MID, HIGH) revealed a main effect of CAPACITY trending towards significance (F_(2,72)_ = 2.88, p = 0.063, $\eta_{p}^{2}$ = 0.07), showing a significant linear trend (high > mid > low, p = 0.029), but no significant interactions.

### Retrospective strategy analysis and other questionnaires

We found no modulation of mood, alertness, sadness or expectations driven either by brain stimulation, strategy instructions or their interaction (see supplemental material for a full description). To validate our strategy manipulation, at the end of day 2, we asked participants to report the strategy used in each run. After strategy instructions, only one participant in the ACTIVE-STRATEGY group failed to use the strategy after the first session, with two participants in the CONTROL-STRATEGY group reporting a failure to adopt the strategy. We also found no difference in the strategy used at baseline between the four groups (X^2^_(30)_ = 29.63, p = 0.484) or between CAPACITIES (X^2^_(20)_ = 21.87, p = 0.348). More details on the strategy questionnaire are reported in the Supplementary material.

### Power analysis

We conducted a power analysis with the program G*Power (Erdfelder, Faul, & Buchner, 1996) to determine if our design had sufficient power to detect a 3-way interaction effect of the same size as the one found here. For the ONLINE effects, the effect size of the three-way interaction contrast was ${\upeta }_{{\text{p}}}^{2}$ = 0.14, assuming a power of 0.80, we would have needed 48 subjects to detect such an effect. For the OFFLINE effects, the effect size of the three-way interaction contrast was ${\upeta }_{{\text{p}}}^{2}$ = 0.07, assuming a power of 0.80, we would have needed 72 subjects to detect such an effect*.*

## Discussion

The present study investigated the impact of strategy instructions to increase the effectiveness of transcranial direct current stimulation on a spatial WM task ^13,42^. Importantly and consistent with our prediction, the results reveal that the combination of strategy and stimulation is particularly beneficial for individuals with initially low working memory capacity.

Individual differences in age and education influence WM baseline abilities ^30,31,35,47^, which in turn impact an individual’s responsiveness to cognitive tasks and the ability to benefit from stimulation induced plasticity. The aptitude-by-treatment interaction theory states that the outcome of treatment is modulated by individual factors. Relevant to the current study, one of these factors is the ability to derive an efficient strategy ^33,47^.

Consistent with the above factors, we found the combination of strategy instructions and tDCS improved WM over and above either strategy or stimulation alone but only for individuals with low baseline capacity. As Lovden et al. suggest ^35^, individuals with high capacity likely have the cognitive resources to devise a strategy and adapt it to increasing task difficulty, whereas low capacity individuals require additional resources to use the strategy provided, which was facilitated by tDCS-induced plasticity. Low capacity individuals benefitted only from the combination of tDCS and strategy instructions, indicating that even when provided with a strategy they may not have had the cognitive resources to use it effectively. tDCS-induced plasticity may facilitate these additional resources. In high capacity individuals, the effect of tDCS was not evident as we speculate they may already have had sufficient cognitive resources to either use the provided instructions effectively or to devise an effective strategy on their own. To summarise, our findings suggest that tDCS acts similarly for both groups, but its effect is beneficial, and ‘measurable’ at the behavioural level, only when additional resources are required to cope with cognitive demand. Further investigations are needed, which would include neurophysiological markers of stimulation effects, and to understand whether our speculations are consistent with the underlying neurophysiology.

Importantly, individuals with low WM capacity maintained the advantage conferred by combined strategy instructions and brain stimulation after the stimulation ended (post-assessment offline session, see Fig. 6)^15^. Our results stand in contrast with those from Jones et al.^48^, who found that WM strategy with tDCS improved performance in individuals with high capacity. There are a few experimental design differences that likely account for the discrepancy between their and our results. First, while we manipulated strategy as a between-participant design, they manipulated it as a within-participant factor. Secondly, Jones et al. used a change detection task, while we used an n-back task, the latter being more likely to necessitate efficient WM updating. Finally, they used different stimulation parameters (5 cm × 7 cm sponge electrodes, 1.5 mA for 10 min targeting left PFC).

We did not find an effect on the visual fixed nback (transfer) task of either strategy instructions or stimulation, although every group improved in the second session with improvement a function of initial capacity. It is worth noting that the strategy instructions could not be applied to the transfer task, therefore individuals would have had to adapt the taught strategy to the new task ^47^. Moreover, the random nature of the stimuli makes them difficult to encode (either verbally or using a specific strategy), therefore participants have likely to rely on imagery, which is a different strategy than the one they have been practising. Also, as Berryhill ^32^ pointed out, often transfer effects are found at follow-up, after a period of no contact, while they are not visible at posttest.

Motivation may play an important role in cognitive performance. The overall effect of STIMULATION and STRATEGY on motivation is detailed in the Supplementary material. To summarize, we found that overall, individuals’ positive attitude (as measured by the PANAS) significantly improved by the end of the experiment, as well as the expectation toward cognitive training and brain stimulation, but we found no significant differences between groups at baseline, overall or within capacity level (ps > 0.5). Furthermore, we investigated if motivation has an impact on how an individual devises a strategy in the first session (when strategy instructions were yet to be provided). We found that, while almost everyone reported to have used a strategy of some sort, there was no correlation (ps > 0.1) between motivation measures (positive attitude, motivation and expectation towards the cognitive training), and performance on the aNback task in the baseline session. Assuming that an effective strategy leads to better performance, we argue that our finding shows that motivation did not modulate strategy effectiveness. Moreover, we visually inspected the relationship between the effectiveness of strategies devised by individuals on their own in the baseline session and their motivation and found no clear relationship between effectiveness and motivation (more details are reported in the Supplementary Material).

Individuals undertaking a cognitive task are likely to devise a strategy they deem efficient. However, the time required to develop a strategy is highly variable and cognitively demanding, potentially nulling the positive effects of brain plasticity ^49^. Manipulating strategy enabled us to investigate the importance of this construct by reducing the variability that arises when participants are left to derive their own. We are confident that participants engaged with the strategy provided, based on feedback questionnaires.

We acknowledge that providing participants with a verbal strategy changes the nature of an otherwise spatial task. However, two considerations are important. First, devising a strategy requires resources that are taken away from the task, as participants use some of the inter-stimulus time to make an effective choice ^50^. Thus, when a strategy is provided, participants can construct a better representation of the visual stimuli, supported by the right DLPFC and further enhanced by stimulation over that region. Second, the right DLPFC has connections with other brain regions ^51–54^, therefore stimulation may augment brain areas subserving verbal working memory, such as the left DLPFC.

A second limitation of our study is the lack of a neurophysiological measure (e.g., EEG) that could support the interpretation of our findings. While we acknowledge the importance of such measures (and we are undertaking a study addressing our research question with EEG), we believe that the present results are informative and provide important insight into the efficacy as well as mechanisms of cognitive training combined with brain stimulation. Neurophysiological measures will further add to the understanding of the brain mechanisms underlying cognitive training in combination with brain stimulation.

Finally, we are aware that the sample size is small, however, both power analysis and the Bayesian analysis support our finding. Power analysis showed that a sample of 72 subjects would have been needed to detect effects of the same size of those found here. Furthermore, Bayesian analysis finds anecdotal to moderate evidence that the model best explaining the data includes both STIMULATION and STRATEGY factors, and their interaction, with the evidence stronger in the LOW CAPACITY individuals.

The present study reveals that initial skill set is linked to task outcome and sets limits on the effectiveness of brain stimulation in combination with cognitive training to improve working memory. Importantly, our findings have implications for training regimens, e.g., by designing interventions predicated on baseline skill set, or interventions focusing on strategy development for specific attentional skills in addition to task repetition. Future research should investigate the impact of strategy and stimulation on the *maintenance* of the benefits gained during a training regimen ^36^. It will also be important to determine the time frame to which stimulation can confer long-term advantage. Moreover, it will be important to understand how strategy instructions, combined with stimulation, may promote performance improvements in other tasks, in both the same and in related cognitive domains. Finally, while we focused on young participants, the interaction of strategy, stimulation, and baseline capacity in older adults remains to be investigated. Evidence shows that older adults use different cognitive resources in a working memory task with respect to young adults ^55,56^, with older adults making a more extensive use of attention, verbal memory and updating than their younger counterparts. It would therefore not be surprising that older adults may benefit even more from strategy instructions than young participants, especially those whose baseline performance is impaired due to normal or abnormal ageing.

## Supplementary Information

Supplementary Information.

## Data Availability

The research protocols used in this research were approved by the ethics committee of the University of Birmingham, Birmingham, UK [ERN_12-1002AP18R] and was conducted in accordance with the University’s Code of Practice for Research. Data will be made available upon reasonable request to the authors.
