# Supplementary material for: Impact of tDCS on working memory training is enhanced by strategy instructions in individuals with low working memory capacity
Source: Sci Rep. 2021 Mar 9;11:5531. doi: 10.1038/s41598-021-84298-3 (PMC7943602; doi:10.1038/s41598-021-84298-3)
Supplement: Supplementary file 1 — Supplementary Information. [file 41598_2021_84298_MOESM1_ESM.pdf]

SUPPLEMENTARY MATERIAL

IMPACT OF TDCS ON WORKING MEMORY TRAINING IS ENHANCED BY STRATEGY INSTRUCTION IN INDIVIDUALS WITH LOW WORKING MEMORY CAPACITY

Sara Assecondi[1,2,,#], Rong Hu[3,1,2,#] , Gail Eskes[4], Xiaoping Pan[3], Jin Zhou[3],Kim Shapiro[1,2]*

[1] Visual Experience Laboratory, School of Psychology, University of Birmingham, UK
[2] Center for Human Brian Health (CHBH), University of Birmingham, UK
[3] Department of Neurology, Guangzhou First People's Hospital, School of Medicine, South China University of Technology, Guangzhou, Guangdong, China
[4] Departments of Psychiatry and Psychology & Neuroscience, Dalhousie University, Halifax, NS, Canada

*Corresponding author email: sara.assecondi@gmail.com
[#]Equally contributed to this work

DEMOGRAPHIC AND BASELINE CHARACTERISTICS

Statistical tests showed that the four groups did not differ in age, gender distribution, years of education, motivation, mood attitude or baseline performance (all ps > 0.05). Demographic characteristics and baseline scores are reported in Table S1. A chi-square test of independence was performed to examine the relation between groups (ACTIVE-STRATEGY, ACTIVE-NoSTRATEGY, SHAM-STRATEGY, SHAM-NoSTRATEGY) and CAPACITY. The relation between these variables was not significant, ($X^2(6, N = 84) = 2.79, p=0.835$), indicating that groups were equally likely to contain individuals with low-, mid- or high-capacity. Another chi-square test of independence showed no significant relation between groups and gender ($X^2(3, N=84) = 2.33, p= 0.506$).

Table S1 Demographic characteristics and descriptive statistics of the overall sample, divided by groups. For each group, we report the count N and the average score, together with its standard error, the F statistics, corresponding p-value and effect size ($\boldsymbol{\eta_p^2}$) from a 1-way ANOVA between groups.

		ACTIVE-STRATEGY	ACTIVE-NoSTRATEGY	SHAM-STRATEGY	SHAM-NoSTRATEGY	F(3,80)	p	$\boldsymbol{\eta_p^2}$
DEMOGRAPHICS	N	21	21	21	21	--	--	--
	Age	21.67 ± 1.23	20.90 ± 0.81	19.10 ± 0.21	20.86 ± 0.79	1.67	0.18	0.06
	Gender (F/M)	14/7	17/4	13/8	16/5	--	--	--
	Years of education	17.05 ± 0.69	15.95 ± 0.86	15.29 ± 0.36	15.57 ± 0.64	1.36	0.26	0.05
MOTIVATION, ATTITUDE AND EXPECTATION	Alertness	3.48 ± 0.16	3.29 ± 0.17	3.29 ± 0.18	3.33 ± 0.17	0.27	0.85	0.01
	Motivation	3.76 ± 0.17	3.67 ± 0.14	3.48 ± 0.19	3.67 ± 0.14	0.54	0.65	0.02
	Sadness	3.67 ± 0.20	3.71 ± 0.22	3.76 ± 0.17	3.62 ± 0.25	0.08	0.97	0.00
	Expectation on CT	3.62 ± 0.16	3.33 ± 0.19	3.29 ± 0.23	3.33 ± 0.20	0.60	0.61	0.02
	Expectation on tDCS	3.38 ± 0.18	2.86 ± 0.21	2.86 ± 0.20	3.05 ± 0.18	1.68	0.18	0.06
	Positive affect	28.10 ± 1.68	29.19 ± 1.48	26.95 ± 1.82	26.14 ± 1.53	0.66	0.58	0.02
	Negative affect	12.667 ± 0.47	12.33 ± 0.84	13.05 ± 0.85	12.29 ± 0.58	0.25	0.86	0.01
BASELINE SCORES	z($\bar{n}$) in aNback	0.26 ± 0.27	-0.30 ± 0.20	-0.07 ± 0.16	0.11 ± 0.23	1.24	0.30	0.04
	z(d') in fNack	-0.01 ± 0.23	0.01 ± 0.22	-0.06 ± 0.25	0.06 ± 0.19	0.04	0.99	0.00
	Composite capacity score	0.13 ± 0.20	-0.15 ± 0.17	-0.06 ± 0.18	0.09 ± 0.18	0.47	0.70	0.02

Table S2 Demographic characteristics and descriptive statistics of low capacity individuals, divided by groups. For each group, we report the count N and the average score, together with its standard error, the F statistics, corresponding p-value and effect size (η_p^2) from a 1-way ANOVA between groups.

LOW CAPACITY		ACTIVE-STRATEGY	ACTIVE-NoSTRATEGY	SHAM-STRATEGY	SHAM-NoSTRATEGY	F(3,24)	p	η_p^2
DEMOGRAPHICS	N	7	9	6	6	--	--	--
	Age	23.86 ± 3.3	20.56 ± 0.78	19.00 ± 0.37	18.83 ± 0.31	1.99	0.14	0.20
	Gender (F/M)	4/3	7/2	6/0	5/1	--	--	--
	Years of education	17.86 ± 1.12	15.22 ± 0.72	15.50 ± 0.34	14.83 ± 0.98	2.52	0.08	0.24
MOTIVATION, ATTITUDE AND EXPECTATION	Alertness	3.29 ± 0.36	3.33 ± 0.24	3.67 ± 0.33	3.17 ± 0.31	0.43	0.74	0.05
	Motivation	3.43 ± 0.37	3.67 ± 0.29	3.83 ± 0.31	3.50 ± 0.34	0.28	0.84	0.03
	Sadness	3.57 ± 0.30	3.67 ± 0.33	4.17 ± 0.31	3.17 ± 0.48	1.14	0.35	0.12
	Expectation on CT	3.71 ± 0.36	3.44 ± 0.29	3.50 ± 0.43	3.50 ± 0.34	0.12	0.95	0.01
	Expectation on tDCS	3.14 ± 0.40	3.56 ± 0.24	3.00 ± 0.37	2.83 ± 0.17	1.10	0.37	0.12
	Positive affect	28.00 ± 3.72	28.67 ± 2.40	25.83 ± 3.39	27.50 ± 4.34	0.13	0.94	0.02
	Negative affect	12.71 ± 0.87	12.11 ± 0.95	14.00 ± 2.02	13.33 ± 1.38	0.41	0.75	0.05
BASELINE SCORES	z($\overline{n}$) in aNback	-0.81 ± 0.21	-1.10 ± 0.13	-0.78 ± 0.24	-0.83 ± 0.23	0.69	0.57	0.08
	z(d') in fNack	-0.96 ± 0.15	-0.72 ± 0.15	-1.42 ± 0.29	-0.93 ± 0.24	2.10	0.13	0.21
	Composite Capacity score	-0.88 ± 0.10	-0.91 ± 0.10	-1.10 ± 0.23	-0.88 ± 0.19	0.44	0.73	0.05

Table S3 Demographic characteristics and descriptive statistics of mid capacity individuals, divided by groups. For each group, we report the count N and the average score, together with its standard error, the F statistics, corresponding p-value and effect size (η_p^2) from a 1-way ANOVA between groups.

MID CAPACITY		ACTIVE-STRATEGY	ACTIVE-NoSTRATEGY	SHAM-STRATEGY	SHAM-NoSTRATEGY	F(3,23)	p	η_p^2
DEMOGRAPHICS	N	6	6	9	6	--	--	--
	Age	20.67 ± 1.89	19.17 ± 0.17	19.00 ± 0.24	23.00 ± 1.86	2.39	0.09	0.24
	Gender (F/M)	5/1	6/0	6/3	5/1	--	--	--
	Years of education	17.83 ± 1.66	14.50 ± 0.43	14.67 ± 0.29	17.67 ± 1.65	2.90	0.06	0.27
MOTIVATION, ATTITUDE AND EXPECTATION	Alertness	4.00 ± 0.326	2.83 ± 0.40	2.67 ± 0.17	3.00 ± 0.37	1.33	0.27	0.07
	Motivation*	4.17 ± 0.31	3.50 ± 0.22	2.89 ± 0.20	3.67 ± 0.33	4.49	0.01	0.37
	Sadness	3.33 ± 0.56	3.83 ± 0.17	3.33 ± 0.17	3.33 ± 0.56	0.42	0.74	0.05
	Expectation on CT	3.67 ± 0.21	2.67 ± 0.33	3.00 ± 0.29	3.33 ± 0.49	1.45	0.25	0.16
	Expectation on tDCS	3.50 ± 0.22	2.17 ± 0.17	2.78 ± 0.32	3.17 ± 0.48	2.73	0.07	0.26
	Positive affect	30.00 ± 2.08	24.33 ± 1.82	26.56 ± 1.76	22.17 ± 2.30	2.54	0.08	0.25
	Negative affect	12.50 ± 1.06	13.33 ± 2.62	13.22 ± 1.39	11.83 ± 1.22	0.17	0.91	0.02
BASELINE SCORES	$z(\bar{n})$ in aNback	0.13 ± 0.36	0.02 ± 0.24	0.01 ± 0.15	-0.18 ± 0.28	0.25	0.86	0.03
	z(d') in fNack	-0.16 ± 0.28	-0.05 ± 0.27	-0.02 ± 0.12	-0.13 ± 0.23	0.09	0.97	0.01
	Composite Capacity score	-0.01 ± 0.07	-0.01 ± 0.09	-0.01 ± 0.08	-0.15 ± 0.07	0.73	0.55	0.09

Table S4 Demographic characteristics and descriptive statistics of high capacity individuals, divided by groups. For each group, we report the count N and the average score, together with its standard error, the F statistics, corresponding p-value and effect size (η_p^2) from a 1-way ANOVA between groups.

HIGH CAPACITY		ACTIVE-STRATEGY	ACTIVE-NoSTRATEGY	SHAM-STRATEGY	SHAM-NoSTRATEGY	F(3,25)	p	η_p^2
DEMOGRAPHICS	N	8	6	6	9	--	--	--
	Age	20.50 ± 1.27	23.17 ± 2.46	19.33 ± 0.56	20.78 ± 1.21	1.01	0.40	0.11
	Gender (F/M)	5/3	4/2	1/5	6/3	--	--	--
	Years of education	15.75 ± 0.88	18.50 ± 2.64	16.00 ± 1.13	14.67 ± 0.58	1.43	0.26	0.15
MOTIVATION, ATTITUDE AND EXPECTATION	Alertness	3.25 ± 0.16	3.67 ± 0.21	3.83 ± 0.31	3.67 ± 0.24	1.18	0.34	0.12
	Motivation	3.75 ± 0.16	3.83 ± 0.17	4.00 ± 0.37	3.78 ± 0.15	0.26	0.85	0.03
	Sadness	4.00 ± 0.19	3.67 ± 0.61	4.00 ± 0.37	4.11 ± 0.31	0.26	0.86	0.03
	Expectation on CT	3.50 ± 0.27	3.83 ± 0.17	3.50 ± 0.56	3.22 ± 0.28	0.57	0.64	0.06
	Expectation on tDCS	3.50 ± 0.27	2.50 ± 0.43	2.83 ± 0.40	3.11 ± 0.26	1.62	0.21	0.16
	Positive affect	26.75 ± 2.80	34.83 ± 1.60	28.67 ± 5.15	27.89 ± 1.31	1.49	0.24	0.15
	Negative affect	12.75 ± 0.73	11.67 ± 0.71	11.83 ± 0.95	11.89 ± 0.63	0.44	0.73	0.05
BASELINE SCORES	z($\bar{n}$) in aNback	1.28 ± 0.40	0.58 ± 0.26	0.53 ± 0.24	0.94 ± 0.29	1.17	0.34	0.12
	z(d') in fNack	0.94 ± 0.28	1.15 ± 0.35	1.25 ± 0.17	0.84 ± 0.09	0.68	0.57	0.08
	Composite Capacity score	1.11 ± 0.16	0.86 ± 0.12	0.89 ± 0.08	0.89 ± 0.13	0.84	0.48	0.09

Overall group differences

<u>ONLINE EFFECTS</u>: We analysed performance changes in training across the two tDCS sessions in the adaptive spatial nBack task (aNback) as the average difference between the mean 'n' within a session (excluding the first block) and the mean 'n' at baseline ($\Delta\bar{n} = \bar{n} - \bar{n}_{\text{baseline}}$). We conducted a 3-way mixed ANOVA with two between-subject factors (STIMULATION: ACTIVE, CONTROL x STRATEGY: STRATEGY, NoSTRATEGY) and one within-subject factor (TIME: change at DAY 1, DAY 2). We found a main effect of TIME ($F_{(1,80)}$=31.72, p<0.001, η_p^2=0.28) and STRATEGY ($F_{(1,80)}$ = 4.60, p = 0.035, η_p^2 = 0.05) and a significant interaction of TIME x STRATEGY ($F_{(1,80)}$ = 4.56, p = 0.036, η_p^2 = 0.05). Follow-up analysis of the interaction effect showed that the STRATEGY group on DAY 2 performed significantly better than on DAY 1 ($t_{(20)}$ = 5.49, p_h < 0.001), and better than the NO-STRATEGY group on DAY 2 ($t_{(20)}$ = 2.74, p_h = 0.03). Also, the NO-STRATEGY group performed better on DAY 2 than on DAY 1 ($t_{(20)}$ = 2.47, p_h = 0.05). To summarize, practice has an overall positive effect, which is larger in the STRATEGY group.

<u>OFFLINE EFFECTS</u>: We analysed the overall changes in performance in the aNback task ($\Delta\bar{n}$), calculated as the change in $\bar{n}$ in the post-assessment in relation to the baseline, using a 2-way independent ANOVA (STIMULATION: ACTIVE, CONTROL x STRATEGY: STRATEGY, NoSTRATEGY). We found a main effect of STRATEGY ($F_{(1,80)}$ = 9.44, p = 0.003, η_p^2 = 0.11), with the STRATEGY group outperforming the NoSTRATEGY group.

<u>TRANSFER EFFECTS</u>: A 2-way independent ANOVA with 2 between-subject factors (STIMULATION: ACTIVE, CONTROL x STRATEGY: STRATEGY, NoSTRATEGY) revealed no significant effects (ps > 0.1).

Possible adverse effects of brain stimulation

Possible adverse effects were collected from participants after each stimulation session, together with the likelihood of such effects being caused by stimulation. Table S5 summarizes the number of participants reporting a side effect, thought to be related to stimulation, with their respective group percentage. There was no difference in participants' group allocation guesses, indicating that subjects were blind to the stimulation group (X^2(1, N=83) = 0.86, p = 0.35)

	ACTIVE [n (%)]		SHAM [n (%)]	
SIDE EFFECT	DAY 1	DAY2	DAY 1	DAY 2
HEADACHE	1 (2)	4 (10)	12 (29)	7 (17)
PAIN IN NECK	4 (10)	2 (5)	0 (0)	1 (2)
ITCHING	25 (60)	18 (43)	24 (57)	18 (42)
SLEEPINESS	11 (26)	21 (50)	18 (43)	17 (40)
TROUBLE CONCENTRATING	14 (33)	19 (45)	18 (43)	15 (36)
ACUTE MOOD CHANGE	3 (7)	4 (10)	6 (14)	5 (12)
FATIGUE	9 (21)	17 (40)	18 (43)	18 (43)
NAUSEA	1 (2)	1 (2)	1 (2)	0 (0)
MUSCLE TWITCH IN FACE OR NECK	4 (10)	2 (5)	2 (5)	1 (2)
TINGLING	37 (88)	33 (79)	36 (86)	35 (83)
BURNING	25 (60)	25 (60)	29 (69)	25 (60)
EPILEPTIC SEIZURE	0 (0)	0 (0)	0 (0)	0 (0)
LIGHT FLASHES	1 (2)	1 (2)	1 (2)	0 (0)
UNCOMFORTABLE FEELING	21 (50)	15 (36)	20 (48)	16 (38)

Table S5 Number n (and percentage %) of subjects in the ACTIVE and CONTROL groups reporting each side effect

MOOD AND ATTITUDE TOWARD THE STIMULATION

Mood and attitude towards the intervention were monitored before each testing session, see Table S6. A 2-way mixed ANOVA (between subjects: STIMULATION: ACTIVE, CONTROL x STRATEGY: STRATEGY, NO STRATEGY; within-subject: TIME: T0,T1 on DAY 1, T2,T3 on DAY 2) revealed no effect on ALERTNESS, MOTIVATION or SADNESS (all ps > 0.1). We did find a main effect of TIME on EXPECTATION ON WM PERFORMANCE ($F_{(3,231)} = 3.24$, $p_{GG} = 0.031$, $n_2 = 0.01$). We also found a significant effect of TIME ($F_{(3,234)} = 9.79$, $p_{GG} < 0.001$, $n_2 = 0.025$), and a significant TIME x STIMULATION x STRATEGY interaction ($F_{(3,234)} = 4.711$, $p_{GG} = 0.009$, $n_2 = 0.01$) on EXPECTATION ON tDCS with the ACTIVE – NO STRATEGY group having higher expectation towards the end of the intervention. We found no effect of the intervention on NEGATIVE ATTITUDE scores (as self-reported on the PANAS). We did find a significant effect of TIME on POSITIVE ATTITUDE scores ($F_{(1,80)} = 8.40$, $p = 0.005$, $n_2 = 0.01$), with scores higher before than after the intervention (Table S7).

		df	F	p	η^2
ALERTNESS	TIME	3,231	1.24	0.296	0.01
	TIME * STRATEGY	3,231	0.38	0.740	< 0.00
	TIME * STIMULATION	3,231	0.58	0.603	< 0.00
	TIME * STRATEGY * STIMULATION	3,231	0.59	0.595	< 0.00
	STRATEGY	1,77	0.22	0.638	< 0.00
	STIMULATION	1,77	1.66	0.201	0.02
	STRATEGY * STIMULATION	1,77	2.07	0.154	0.03
MOTIVATION		df	F	p	η^2
	TIME	3,228	2.54	0.063	0.01
	TIME * STIMULATION	3,228	0.39	0.741	< 0.00
	TIME * STRATEGY	3,228	0.79	0.488	< 0.00
	STIMULATION	1,76	1.18	0.281	0.01
	STRATEGY	1,76	0.32	0.575	< 0.00
	STIMULATION * STRATEGY	1,76	0.81	0.371	0.01
	TIME * STIMULATION * STRATEGY	3,228	0.14	0.925	< 0.00
SADNESS		df	F	p	η^2_p
	TIME	3,231	0.06	0.949	< 0.00
	STRATEGY	1,77	0.24	0.624	< 0.00
	STIMULATION	1,77	0.06	0.814	< 0.00
	TIME * STIMULATION	3,231	0.42	0.673	< 0.00
	TIME * STRATEGY	3,231	0.15	0.874	< 0.00
	STIMULATION * STRATEGY	1,77	0.00	0.979	< 0.00
	TIME * STIMULATION * STRATEGY	3,231	0.60	0.563	< 0.01
EXPECTATION ON WM PERFORMANCE		df	F	p	η^2
	TIME **	3,231	3.24	0.031	0.01
	STIMULATION	1,77	2.97	0.089	0.04
	STRATEGY	1,77	0.14	0.708	< 0.00
	TIME * STIMULATION	3,231	1.47	0.229	< 0.00
	TIME * STRATEGY	3,231	0.48	0.663	< 0.00
	STIMULATION * STRATEGY	1,77	0.36	0.551	< 0.00
	TIME * STIMULATION * STRATEGY	3,231	1.99	0.127	< 0.00
EXPECTATION ON BRAIN STIMULATION		df	F	p	η^2
	TIME **	3,234	9.79	**< .001**	0.02
	STIMULATION	1,78	3.28	0.074	0.04
	STRATEGY	1,78	0.24	0.624	< 0.00
	TIME * STIMULATION	2,111	2.20	0.111	0.01
	TIME * STRATEGY	2,111	1.42	0.243	< 0.00
	STIMULATION * STRATEGY	1,78	0.01	0.939	< 0.00
	TIME * STIMULATION * STRATEGY **	2,111	4.71	**0.009**	0.01

*Table S6 Output of a 3-way mixed ANOVA (between subjects: STIMULATION: ACTIVE, CONTROL x STRATEGY: STRATEGY, NO STRATEGY; within-subject: TIME: T0, T1 on day 1, T2,T3 on day 2) on attitude and expectation, significant finding are marked with **.*

		df	F	p	η^2
POSITIVE AFFECT	TIME **	1,80	8.40	**0.005**	< 0.01
	STRATEGY	1,80	0.08	0.776	< 0.00
	STIMULATION	1,80	2.83	0.096	< 0.03
	TIME ✻ STRATEGY	1,80	0.28	0.600	< 0.00
	TIME ✻ STIMULATION	1,80	1.27	0.262	< 0.00
	STRATEGY ✻ STIMULATION	1,80	0.09	0.765	< 0.00
	TIME ✻ STRATEGY ✻ STIMULATION	1,80	0.51	0.477	< 0.00
NEGATIVE AFFECT		df	F	p	η^2
	TIME	1,80	0.74	0.392	< 0.00
	STRATEGY	1,80	0.20	0.654	< 0.00
	STIMULATION	1,80	0.07	0.794	< 0.00
	TIME ✻ STRATEGY	1,80	0.40	0.530	< 0.00
	TIME ✻ STIMULATION	1,80	0.00	1.000	< 0.00
	STRATEGY ✻ STIMULATION	1,80	0.07	0.794	< 0.00
	TIME ✻ STRATEGY ✻ STIMULATION	1,80	0.01	0.909	< 0.00

*Table S7 A 3-way mixed ANOVA (between subjects: STIMULATION: ACTIVE, CONTROL x STRATEGY: STRATEGY, NO STRATEGY; within-subject: TIME) on the PANAS, significant findings are marked with **.*

RETROSPECTIVE STRATEGY QUESTIONNAIRE

In order to analyze self-reported feedback on strategy use, the following questions were asked at the end of the second day of testing. The STRATEGY group was asked if they used strategy explained to them at the beginning of the experiment, while the No STRATEGY group was asked if they used a strategy and how they would classify it according to the following. In between brackets we report how each strategy was classified by us in the following analysis.

- (REHEARSAL) I repeated the location of the target in my mind
- (GROUPING) I remembered the location of target in groups (e.g. 2 or more location together)
- (GROUPING) I created a group of location in my mind and dropped the last location when a new location appeared
- (GROUPING) I split the location into different series, and compared those to each other
- (ASSOCIATION) I used the meaning of the target to remember or connect them (such as top left, top middle, top right)
- (IMAGERY) I connected the location of targets with lines
- (ASSOCIATION) I thought about other things that could relate to the location (such as the time of the clock)
- (IMAGERY) I created a visual image based on the location

- (CONCENTRATION) I simply concentrated on the location of target
- (ASSOCIATION) I transfer the location of targets as numbers, such as 1,2,3,6,7,8,9
- (ASSOCIATION+GROUPING) I transfer the location of targets as numbers, such as 1,2,3,6,7,8,9, split the numbers into different sets, and compared those to each other,
- (LOOK) I pictured the way the target looked on the screen
- (FAMILIARITY) I answered based on what location seemed recent or familiar
- (CHECKLIST) I expected certain location to appear and mentally checked them off as they arrived
- (INTUITION) I just use my intuition
- (UNSPECIFIED) Other (Please describe below)

We asked ourselves if motivation could be a factor for an individual in developing a more efficient strategy on their own. We followed this up with two additional analysis:

1) Correlation between motivation and performance. Following the assumption that using a more effective strategy would lead to better performance, we analysed the correlation between performance at baseline (as the average n at baseline on the aNback) and motivation (as measured by either Positive Attitude (PANAS), expectation towards Cognitive training and General motivation (both measured on a Likert scale). All correlations were not significant (ps > 0.1). Therefore, more motivated individuals were not more likely to develop an effective strategy than less motivated individuals.
2) Correlation between strategy effectiveness and motivation. At baseline, before being instructed with a strategy, it is possible that individuals would still develop a strategy on their own. We collected this information during the experiment (see STRATEGY questionnaire in the Supplementary Material), however, the following analysis could only be qualitative as this kind of feedback is not very reliable (individuals often have difficulties verbalising the strategy used). First, we classified the strategy reported by the participant as EFFECTIVE (those similar to the one provided by us, e.g., ASSOCIATION + GROUPING) or INEFFECTIVE (different from the one provided by us), then we visualised the distribution of motivation scores across the two strategy categories (see here below) and found that there was no visual relationship between strategy effectiveness and motivation.

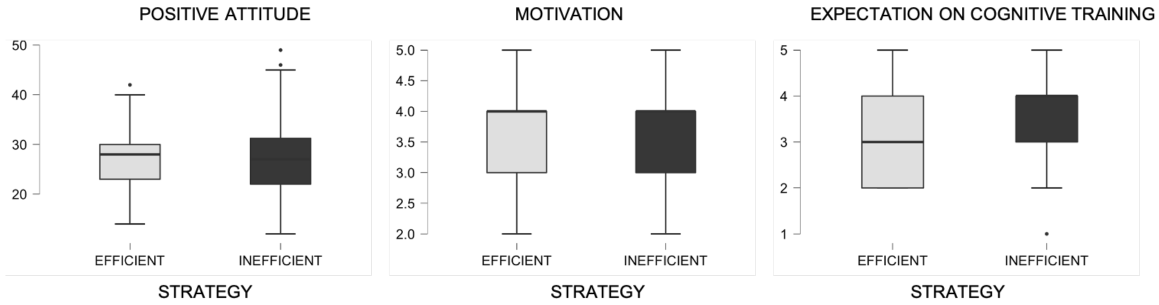

Figure S1 Qualitative comparison of motivation as a function of strategy efficacy.
